# Vitamin D status of children with paediatric inflammatory multisystem syndrome temporally associated with severe acute respiratory syndrome coronavirus 2 (PIMS-TS)

**DOI:** 10.1017/S0007114521001562

**Published:** 2022-03-28

**Authors:** Angeline Darren, Meissa Osman, Kavitha Masilamani, Syed Habib Ali, Hari Krishnan Kanthimathinathan, Ashish Chikermane, Eslam Al-Abadi, Steven B. Welch, Scott Hackett, Barnaby R. Scholefield, Suma Uday, Deepthi Jyothish

**Affiliations:** 1 Department of General Paediatrics, Birmingham Women’s and Children’s NHS Foundation Trust, Birmingham, UK; 2 Paediatric Intensive Care Unit, Birmingham Women’s and Children’s NHS Foundation Trust, Birmingham, UK; 3 Department of Cardiology, Birmingham Women’s and Children’s NHS Foundation Trust, Birmingham, UK; 4 Childhood Arthritis and Rheumatic Diseases Unit, Birmingham Women’s and Children’s NHS Foundation Trust, Birmingham, UK; 5 Department of Paediatrics, Birmingham Chest Clinic and Heartlands Hospital, University Hospitals Birmingham, Birmingham, UK; 6 Birmingham Acute Care Research Group, Institute of Inflammation and Ageing, University of Birmingham, Birmingham, UK; 7 Department of Endocrinology and Diabetes, Birmingham Women’s and Children’s NHS Foundation Trust, Birmingham, UK

**Keywords:** Coronavirus disease 2019, Paediatric inflammatory multisystem syndrome temporally associated with severe acute respiratory syndrome coronavirus 2, Multisystem Inflammatory Syndrome in Children, Paediatric intensive care unit, Severe acute respiratory syndrome coronavirus 2, Vitamin D deficiency

## Abstract

Coronavirus disease 2019 (COVID-19) has caused mild illness in children, until the emergence of the novel hyperinflammatory condition paediatric inflammatory multisystem syndrome temporally associated with severe acute respiratory syndrome coronavirus 2 (SARS-CoV-2) (PIMS-TS). PIMS-TS is thought to be a post-SARS-CoV-2 immune dysregulation with excessive inflammatory cytokine release. We studied 25 hydroxyvitamin D (25OHD) concentrations in children with PIMS-TS, admitted to a tertiary paediatric hospital in the UK, due to its postulated role in cytokine regulation and immune response. Eighteen children (median (range) age 8·9 (0·3-14·6) years, male = 10) met the case definition. The majority were of Black, Asian and Minority Ethnic (BAME) origin (89 %, 16/18). Positive SARS-CoV-2 IgG antibodies were present in 94 % (17/18) and RNA by PCR in 6 % (1/18). Seventy-eight percentage of the cohort were vitamin D deficient (< 30 nmol/l). The mean 25OHD concentration was significantly lower when compared with the population mean from the 2015/16 National Diet and Nutrition Survey (children aged 4–10 years) (24 *v*. 54 nmol/l (95 % CI −38·6, −19·7); *P* < 0·001). The paediatric intensive care unit (PICU) group had lower mean 25OHD concentrations compared with the non-PICU group, but this was not statistically significant (19·5 *v*. 31·9 nmol/l; *P* = 0·11). The higher susceptibility of BAME children to PIMS-TS and also vitamin D deficiency merits contemplation. Whilst any link between vitamin D deficiency and the severity of COVID-19 and related conditions including PIMS-TS requires further evidence, public health measures to improve vitamin D status of the UK BAME population have been long overdue.

The coronavirus disease 2019 (COVID-19) pandemic caused by severe acute respiratory syndrome coronavirus 2 (SARS-CoV-2) has caused mild illness in the majority of children, with few severe cases requiring hospitalisation and very few deaths^(1)^, a finding also reflected in data from our paediatric unit^(2)^. However, since April 2020, there has been increasing numbers of children presenting with a hyperinflammatory condition described by the UK Royal College of Paediatrics and Child Health as paediatric inflammatory multisystem syndrome temporally associated with severe acute respiratory syndrome coronavirus 2 (PIMS-TS)^(3)^ and as Multisystem Inflammatory Syndrome in Children associated with COVID-19 by the Centers for Disease Control^(4)^ in the USA. PIMS-TS has overlapping features with other childhood inflammatory conditions such as Kawasaki disease, toxic shock syndrome and macrophage activation syndrome^(5)^ but remains a separate clinical entity with distinct cytokine profiles, including marked elevations in IL-6, IL-8 and IL-10. Recent studies suggest that PIMS-TS is a post-infectious hyperinflammatory syndrome with the putative cause being immune dysregulation by SARS-CoV-2, as evidenced by laboratory confirmation of preceding SARS-CoV-2 infection^(6)^.

The influence of 25 hydroxyvitamin D (25OHD) in PIMS-TS is proposed to be through its well-established role in the modulation of adaptive and innate immunity, including regulation of inflammatory cytokine release^(7)^. 25OHD down-regulates type 1 T cells and up-regulates type 2 T cells by reducing production of pro-inflammatory cytokines (IL-6, IL-8 and IL-17) and increasing anti-inflammatory cytokines (IL-10)^(8)^ and this immunoregulatory action is postulated to be involved in PIMS-TS.

A handful of studies have evaluated the association between 25OHD levels and COVID-19 in adults which were recently summarised in the rapid National Institute for Health and Care Excellence evidence summary^(9)^. To date, no studies have reported the vitamin D status of children with COVID-19 or associated conditions. We report, for the first time, the vitamin D status of children with PIMS-TS admitted to a single tertiary paediatric hospital in the Midlands region of the UK.

## Aims

- Report 25OHD concentrations in children with PIMS-TS requiring hospitalisation.
- Compare mean 25OHD concentrations in children with PIMS-TS to healthy paediatric population.
- Compare 25OHD concentrations and inflammatory markers in children with severe illness with circulatory shock requiring paediatric intensive care unit (PICU group) support to children who experienced a less severe disease course (non-PICU group).

## Methods

### Study design and participants

We undertook a single-centre observational study of children admitted to Birmingham Children’s Hospital, Birmingham, UK, with PIMS-TS, between 12th April and 25th June 2020. All children who met the Royal College of Paediatrics and Child Health^(3)^, Centers for Disease Control ^(4)^, and WHO^(10)^ case definitions of PIMS-TS as detailed in Box 1 were included. Samples for 25OHD concentrations were obtained at presentation in all children as part of diagnostic bloods as per the Royal College of Paediatrics and Child Health PIMS-TS protocol.

**Box 1.** Case definitions of Paediatric inflammatory multisystem syndrome as per RCPCH, CDC and WHO.

RCPCH case definition (3)
1. A child or young person presenting with fever, evidence of inflammation (neutrophilia, elevated CRP and lymphopenia) with evidence of single or multi-organ failure (shock, cardiac, respiratory, renal, gastrointestinal or neurological disorder) with additional features that may include full or partial criteria for Kawasaki’s disease AND
2. Exclusion of any other microbial cause, including bacterial sepsis, staphylococcal or streptococcal shock syndromes, infections associated with myocarditis such as enterovirus AND
3. SARS-CoV-2-PCR testing may be positive or negative.
CDC case definition (4)
1. An individual aged <21 years presenting with fever, laboratory evidence of inflammation and evidence of clinically severe illness requiring hospitalisation, with multisystem (>2) organ involvement (cardiac, renal, respiratory, haematologic, gastrointestinal, dermatologic or neurological AND
2. No plausible alternative diagnosis AND
3. Positive for current or recent SARS-Cov-2 infection by RT-PCR, serology or antigen test, or COVID-19 exposure within the 4 weeks prior to the onset of symptoms.
WHO case definition (11)
1. Children and adolescents 0–19 years of age with fever > 3 days and two of the following: • Rash or bilateral non-purulent conjunctivitis or mucocutaneous inflammation signs (oral, hands or feet). • Hypotension or shock. • Features of myocardial dysfunction, pericarditis, valvulitis or coronary abnormalities (including ECHO findings or elevated troponin/NT-proBNP), • Evidence of coagulopathy (by PT, PTT, elevated D-dimers). • Acute gastrointestinal problems (diarrhoea, vomiting or abdominal pain) AND
2. Elevated markers of inflammation such as ESR, C-reactive protein or procalcitonin AND
3. No other obvious microbial cause of inflammation, including bacterial sepsis, staphylococcal or streptococcal shock syndromes AND
4. Evidence of COVID-19 (RT-PCR, antigen or serology positive), or likely contact with patients with COVID-19.

### Ethics and consent

The project was approved as service evaluation by our institution’s audit committee. Additionally, all parents/legal guardians provided signed informed consent for inclusion of de-identified data in this report. Research Ethics Committee or Health Research Authority approval was not required as per the Health Research Authority assessment tool.

### Data collection

Epidemiological, clinical and laboratory data on all children with PIMS-TS were gathered from clinical notes and electronic health record systems. Ethnicity details were recorded in clinical systems in accordance with the Office of National Statistics classification^(11)^.

### Laboratory methods

SARS-CoV-2 RNA was tested using semi-quantitative RT-PCR from the upper airway using combined nasopharyngeal swabs. Lower airway samples were used in the invasively ventilated children.

Antibody assay to SARS-CoV-2 spike glycoprotein was undertaken using an ELISA test.

25OHD concentrations were measured by quantitative liquid chromatography tandem Mass Spectrometry (AB Sciex API4000 MS/MS’ analyser). The laboratory is subject to External Quality Assurance and meets the requirement of the UK National External Quality Assessment Service vitamin D scheme. The inter- and intra-assay CV were < 10 %.

### Body mass index

Children with BMI centile > 98th were classed as obese and those between 91st and 98th were classed as overweight, in accordance with the Royal College of Paediatrics and Child Health guidelines^(12)^.

### Vitamin D status and reference 25 hydroxyvitamin D concentration data

Based on the Institute of Medicine classification^(13)^, 25OHD concentrations below 50 nmol/l (20 ng/l) were classed as suboptimal. 25OHD concentrations below 30 nmol/l (12 ng/l) were deficient, above 50 nmol/l were sufficient and 30–50 nmol/l were insufficient.

As the children were previously fit and well with no co-morbidities, 25OHD levels were compared with healthy population means. Reference 25OHD concentrations for healthy children were obtained from the nationally representative National Diet and Nutrition Survey data^(14–16)^.

### Statistical analysis

Descriptive statistics were used to describe the baseline characteristics and are reported as numbers (percentages), mean and standard deviation or median and range as appropriate.

A one sample *t* test was used to compare the mean 25OHD concentrations in the study cohort to the paediatric population mean.

An independent *t* test was used to compare normally distributed data (erythrocyte sedimentation rate and C-reactive protein) and a Mann–Whitney *U* test to compare non-normally distributed data (25OHD concentrations) between PICU and non-PICU groups. A *P* value below 0·05 was considered significant. All analyses were performed using SPSS statistical software v25.0 (IBM).

## Results

### Baseline characteristics

Eighteen children with a median (range) age of 8·9 (0·3–14·6) years met the case definition. The majority were males (*n* 10).

A high proportion of the cohort was of Black, Asian and Minority Ethnic (BAME) background (89 %, 16/18). Only two children were of White British ethnicity. Obesity was noted in 31 % (5/16) of the cohort and overweight in 31 % (5/16).

SARS-CoV-2 IgG antibodies were positive in 94 % (17/18) and RNA by PCR was positive in 6 % (1/18). There were no notable co-morbidities, such as liver disease, renal disease, immunodeficiency or other conditions predisposing to recurrent infections and none were on any regular medications.

There were no deaths in the study population.

Details of the clinical characteristics and management are presented in Table 1.

**Table 1.** Baseline characteristics, investigations and treatment of children presenting with paediatric multisystem inflammatory syndrome temporally associated with multisystem inflammatory syndrome in children (PIMS-TS)

Decimal age (years)	Sex	Ethnicity	BMI*	Centile	Respiratory support IV†/NIV‡/HFNC§/O2||	Treatment IVIG¶, MP**	LVEF†† modified Simpson’s method	Laboratory results (reference values, where applicable, are provided in the footnote)				
	F = female; M = Male							Sars-Cov-2	25OHD‡‡ (nmol/l)	Bone profile§§	Inflammatory markers||||	Hb¶¶
PICU												
14·5	F	Black Caribbean	18**·**5	25th–50th)	O2	IVIG, MP	43 %	PCR –ve IgG +ve	19	Adj Ca 2·33 PO4 0·99 ALP 76 (50–150)	CRP 252 ESR 90	79
9·6	M	Asian	20**·**7	91st–98th	HFNC, 02	IVIG, MP	50 %	PCR –ve IgG +ve	8**·**6	Adj Ca N/A*** PO4 N/A ALP 144 (80–330)	CRP 208 ESR 33	68
8·8	F	Black/African Caribbean	22**·**7	98th–99**·**6th	HFNC, 02	IVIG, MP	32 %	PCR –ve IgG +ve	7**·**8	Adj Ca 2·40 PO4 1·67 ALP 70 (80–330)	CRP 422 ESR 90	84
12·6	F	Black/African Caribbean	15**·**2	2nd–9th	None	IVIG	42 %	PCR –ve IgG +ve	17**·**5	Adj Ca 2·25 PO4 1·08 ALP 70 (80–330)	CRP 128 ESR N/A	94
12·7	M	Mixed White/African	19**·**8	75th–91st	O2	IVIG	38 %	PCR –ve IgG +ve	18**·**8	Adj Ca 2·24 PO4 0·95 ALP 76(90–290)	CRP 86 ESR 30	104
5·3	M	Mixed White/African	17**·**9	91st–98th	IV, 02	IVIG	50 %	PCR –ve IgG +ve	16**·**8	Adj Ca 2·32 PO4 1·44 ALP 69 (80–330)	CRP 101 ESR 105	67
10·7	F	Black/African Caribbean	32**·**3	> 99·6th	IV, NIV, 02	IVIG, MP, Tocilizumab	25 %	PCR –ve IgG +ve	21**·**3	Adj Ca 2·54 PO4 1·34 ALP 79 (80–310)	CRP 480 ESR 170	83
8	F	Black/African Caribbean	19**·**9	91st–98th	IV	IVIG, MP, Infliximab	48 %	PCR –ve IgG +ve	30**·**1	Adj Ca 2·37 PO4 1·06 ALP 95 (80–330)	CRP 321 ESR 42	75
8	F	Black/African Caribbean	14**·**3	9th–25th	None	IVIG, MP	50 %	PCR –ve IgG +ve	27	Adj Ca 2·48 PO4 1·05 ALP 120(80–330)	CRP 48 ESR > 170	88
7·1	M	Black/African Caribbean	23**·**4	98th–99**·**6th	HFNC, O2	IVIG, Prednisolone	42 %	PCR –ve IgG +ve	20**·**7	Adj Ca 2·30 PO4 0·8 ALP 102 (80–330)	CRP 264 ESR 12	93
8·0	M	Asian	14**·**7	9th–25th	HFNC	None	28 %	PCR –ve IgG +ve	38	Adj Ca 2·40 PO4 1·17 ALP 59 (80–330)	CRP 244 ESR 45	79
7·0	M	Asian	13**·**9	2nd–9th	IV, HFNC	None	49 %	PCR –ve IgG +ve	13**·**3	Adj Ca 2·37 PO4 1·77 ALP 95(80–330)	CRP 164 ESR 54	84
Non PICU												
13·5	F	Any other White/Romanian	25**·**6	91st–98th	None	IVIG, MP	> 55 %	PCR –ve IgG +ve	12**·**8	Adj Ca 2·39 PO4 1·17 ALP 93 (65–240)	CRP 128 ESR 70	83
7·7	M	Asian	20**·**5	98th–99**·**6th	None	IVIG, MP	> 55 %	PCR –ve IgG +ve	14**·**8	Adj Ca 2·25 PO4 0·98 ALP 107(80–330)	CRP 138 ESR 115	93
9·7	M	Mixed- White/African	17**·**9	91st–98th	None	IVIG	> 55 %	PCR +ve IgG +ve	32**·**8	Adj Ca 2·33 PO4 1·19 ALP 91 (80–330)	CRP 42 ESR 75	76
0·6	F	White	Not available		None	IVIG	> 55 %	PCR –ve IgG –ve	55**·**6	Adj Ca 2·74 PO4 1·77 ALP 148 (80–330)	CRP 92 ESR 82	89
13·2	M	White	27**·**5	98th–99**·**6th	None	None	> 55 %	PCR –ve IgG +ve	65	Adj Ca 2·48 PO4 1·72 ALP 106 (90–290)	CRP 152 ESR 60	136
0·29	M	Black/African Caribbean	Not available		None	None	> 55 %	PCR –ve IgG +ve	10**·**6	Adj Ca 2·48 PO4 1·29 ALP 403 (80–330)	CRP 58 ESR 5	82

PICU, paediatric intensive care unit; 25OHD, 25 hydroxyvitamin D; CRP, C-reactive protein; LVEF, left ventricular ejection fraction.

* BMI centile, RCPCH chart (< 2nd centile low BMI, > 91st overweight, > 98th obese, > 99·6th severely obese).

† Invasive ventilation.

‡ Non-invasive ventilation.

§ High flow nasal cannula oxygen.

|| Supplemental oxygen.

¶ Intravenous Ig.

** Methylprednisolone.

†† Left ventricular ejection fraction (mild impairment 45–54 %, moderate 30–44 % and severe < 30 %).

‡‡ 25-hydroxyvitamin D (deficiency < 30 nmol/l, insufficiency 30–50 nmol/l, sufficiency > 50 nmol/l).

§§ Adjusted Ca (normal range 2·20–2·70 mmol/l), phosphate 1·30–2·40 mmol/l, alkaline phosphatase (age- and sex-specific ranges provided in the table).

|||| C-reactive protein (normal range 0–10 mg/l), erythrocyte sedimentation rate (normal range 0–9 mm/h).

¶¶ Hb (reference range: 3 months–4 years: 110–140 g/l, 5–12 years: 115–140 g/l).

*** Not available.

### Vitamin D status

Based on the Institute of Medicine classification^(13)^, 89 % (16/18) had suboptimal 25OHD concentrations of whom 72 % (13/18) had deficient levels and 17 % (3/18) insufficient levels. Sufficient 25OHD concentrations were noted in only two individuals (11 %, 2/18) who were both of White British ethnicity. None of the BAME children had sufficient 25OHD concentrations and 81 % (13/16) had vitamin D deficiency. Three children (20 %, 3/15) were on commercially available vitamin D supplements purchased over the counter. All children requiring PICU care had suboptimal 25OHD concentrations.

Based on the UK Scientific Advisory Committee on Nutrition recommendations where 25OHD levels < 25 nmol/l are considered inadequate^(17)^, nearly 67 % (13/18) of the cohort were deficient.

### Mean 25 hydroxyvitamin D concentrations

The mean 25OHD concentration of the whole cohort was 23·6 (sd 15·8) nmol/l. The mean 25OHD concentration of the BAME cohort (*n* 16) was 19·0 (sd 8·8) nmol/l and the White cohort (*n* 2) was 60·3 (6·6) nmol/l. 25OHD below 50 nmol/l (20 ng/l) is classed as suboptimal, below 30 nmol/l (12 ng/l) is classed as deficient and 30–50 nmol/l is insufficient.

The mean 25OHD concentrations of the whole cohort were significantly lower when compared with the 2014/2015–2015/2016 National Diet and Nutrition Survey mean 25OHD for children (*n* 514, males = 276) aged 4–10 years^(14)^ (24 *v*. 54 nmol/l (95 % CI −38·6,–19·7); *P* < 0·001).

The mean 25OHD concentration in the BAME group was significantly lower when compared with the mean 25OHD in non-White children (4–18 years, *n* 99/1102) from the 1997–1998 National Diet and Nutrition Survey data^(15)^ (19 *v*. 32 nmol/l (95 % CI −17·6, −8·3); *P* < 0·001).

### Paediatric intensive care unit *v.* non-paediatric intensive care unit group

#### Paediatric intensive care unit group

The majority of patients presented with features of circulatory shock and required PICU care (67 %, 12/18) for circulatory support. All children in the PICU group required inotropes/vasopressors, 33 % (4/12) required invasive ventilation and 8 % (1/12) required hemofiltration for renal failure. None of the children required extracorporeal membrane oxygenation. The median (range) 25OHD concentration in the group was 18·2 (7·8–38) nmol/l. All except two patients had vitamin D deficiency (25OHD < 30 nmol/l). The median (range) C-reactive protein was 226 (48–480) mg/l and erythrocyte sedimentation rate was 54 (12–170) mm/h.

#### Non-paediatric intensive care unit group

Approximately, a third of the study cohort (33 %, 6/18) presented with milder disease features and did not require admission to PICU. The median (range) 25OHD concentration in the group was 23·5 (10·6–65·0) nmol/l. The median (range) C-reactive protein and erythrocyte sedimentation rate were 110 (42–152) mg/l and 67·8 (5–115) mm/h, respectively. There was no mortality in either group.

Investigations and management of PICU group *v*. non-PICU group are presented in Table 2.

**Table 2.** Investigations and management of PICU group *v*. non-PICU group

	PICU		Non-PICU	
	*n*	%	*n*	%
25OHD*				
Median	18·2		23·5	
Range	7·8–38		10·6–65	
CRP†				
Median	226		110	
Range	48–480		42–152	
ESR‡				
Median	54		67·8	
Range	12–170		5–115	
Inotropes/vasopressors	12	100	0	0
Invasive ventilation	4	33	0	0
Hemofiltration	1	8	0	0
LVEF§ normal	0	0	3	50
LVEF mild impairment	4	34	3	50
LVEF moderate impairment	6	50		0
LVEF severe impairment	2	16		0

PICU, paediatric intensive care unit; 25OHD, 25 hydroxyvitamin D; CRP, C-reactive protein; ESR, erythrocyte sedimentation rate; LVEF, left ventricular ejection fraction.

* 25-hydroxyvitamin D (deficiency < 30nmol/l, insufficiency 30–50 nmol/l, sufficiency > 50 nmol/l).

† C-reactive protein (normal range 0–10 mg/l).

‡ Erythrocyte sedimentation rate (normal range 0–9 mm/h).

§ Left ventricular ejection fraction (mild impairment 45–54 %, moderate 30–44 % and severe < 30 %).

**Fig. 1.**
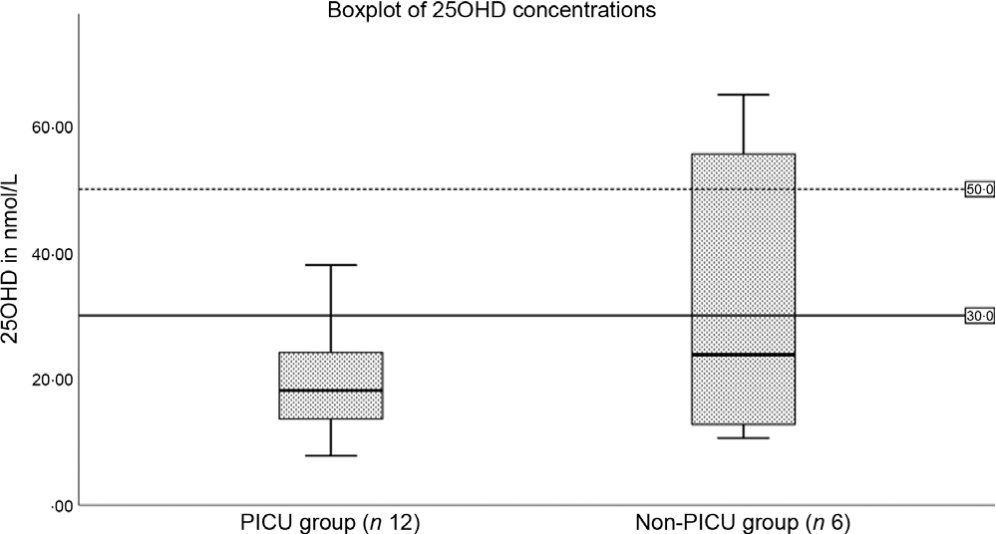
25 hydroxyvitamin D (25OHD) concentrations in paediatric intensive care unit (PICU) and non-PICU groups (*P* = 0·11).

When compared with the non-PICU group, the PICU group had lower mean 25OHD concentrations (Fig. 1) (31 (sd 23·8) *v*. 19·5 (sd 8·8) nmol/l, respectively; *P* = 0·5) but this was not statistically significant. The mean C-reactive protein in the PICU group was significantly higher than that in the non-PICU group (228·5 (sd 131·0) *v*. 101·7 (sd 45·0) mg/l, respectively; *P* = 0·03). The erythrocyte sedimentation rate in the PICU and non-PICU groups did not differ significantly (76·5 (sd 54·4) *v*. 67·8 (sd 36·0) mm/h, respectively; *P* = 0·70).

### Cardiac function

All PICU patients had abnormal (< 55 %) left ventricular ejection fraction based on modified Simpson’s method. Severe impairment was recorded in 16 % (2/12), 50 % (6/12) had moderate impairment and 34 % (4/12) mild impairment.

In the non-PICU group, 50 % (3/6) had normal left ventricular ejection fraction and it was mildly impaired in 50 % (3/6).

Echocardiogram in children with suboptimal 25OHD (16/18) showed normal coronaries in 31 % (*n* 5), prominent coronaries in 63 % (*n* 10) and fusiform dilatation in 6 % (*n* 1). Children with sufficient 25OHD levels (2/18) had normal coronaries and left ventricular ejection fraction and did not require PICU admission.

## Discussion

This is the first report detailing the vitamin D status of children with PIMS-TS requiring hospitalisation, highlighting the alarmingly high proportion of deficiency. PIMS-TS was predominant in children of BAME origin, with 81 % being vitamin D deficient. A high proportion of the whole cohort (72 %) and specifically PICU cohort (83 %) were vitamin D deficient. Children with PIMS-TS had significantly lower mean 25OHD concentrations compared with the nationally representative healthy White British and BAME children; however, there was no association with disease severity.

Suboptimal vitamin D status has been reported in around 50 % of critically ill children in a previous systematic review and meta-analysis^(18)^. We, however, noted suboptimal 25OHD levels in 100 % of our PICU group, probably due to the higher proportion of BAME individuals. The limited National Diet and Nutrition Survey data on BAME individuals in the UK also report a higher proportion of vitamin D deficiency (42·9 %; *n* 63) in comparison with White children (15·0 %, *n* 448)^(16)^. It is noteworthy that all children of BAME origin with PIMS-TS had suboptimal vitamin D status. A higher prevalence of PIMS-TS with disproportionate number of intensive care admissions of children from BAME background has been reported in the UK^(5,19)^ Similarly, hypocalcaemic complications of vitamin D deficiency^(20)^, including nutritional rickets^(21)^, are also 90–166-fold higher in UK Black and Asian children when compared with their White counterparts^(22)^.

The increased risk of acute viral respiratory infections with vitamin D deficiency and the potential protective effects of supplementation has been extensively reported^(23)^. Nonetheless, currently there remains insufficient evidence to recommend routine supplementation to prevent acute respiratory tract infections^(24)^ or COVID-19^(9)^. Evidence on the link between 25OHD status and SARS-CoV-2 continues to emerge, with suggestion of an inverse relationship between circulating 25OHD levels and SARS-CoV-2 positivity^(25)^. The relevance of vitamin D as a modifiable risk factor in severe PIMS-TS due to its actions on unregulated cytokine response requires further consideration. Consistent evidence of PIMS-TS being mediated by amplified inflammatory responses to SARS-CoV-2 and the regulatory actions of 25OHD on pro-inflammatory cytokine signalling further substantiates the possible role of 25OHD in PIMS-TS. This hypothesis is strengthened by the association of low 25OHD with Kawasaki’s disease^(26)^, a childhood inflammatory disease with which PIMS-TS shares considerable overlap^(27)^. 25OHD levels in Kawasaki’s disease have previously been linked to coronary outcomes which may be of relevance in children affected with PIMS-TS^(26)^. Given that nearly 14 % of the UK population are of BAME origin and are at a high risk of vitamin D deficiency^(28)^, ongoing evaluation of the putative link between ethnicity, severe COVID-19 and vitamin D deficiency is essential. Randomised controlled trials in the community would be required to establish a causative link between vitamin D deficiency and COVID-19.

There was no link between 25OHD concentrations and disease severity in our cohort, but our study was not adequately powered to evaluate such a relationship. There is currently insufficient evidence to recommend vitamin D for prevention or treatment of COVID-19 or related conditions. Nonetheless, the finding of widespread deficiency in the BAME population cannot be disregarded. Suboptimal vitamin D status in UK residents^(29)^, especially its BAME residents, is a long-standing problem^(28,30)^ which warrants intervention. Older children and adolescents where there is a higher prevalence of severe COVID-19 and PIMS-TS are also reported to be at a higher risk of vitamin D deficiency^(15,16)^. A number of factors need addressing to improve the vitamin D status of the UK population. Firstly, the threshold for sufficiency in the UK is set as ≥ 25 nmol/l^(17)^, whereas other institutes^(13)^ and consensus guidance^(31)^ regard levels > 50 nmol/l as sufficiency and < 30 nmol/l as deficiency. Secondly, the recommended vitamin D requirement for children > 1 year in the UK is lower (10 µg or 400 IU daily) than that supported by other evidence-based guidance (15 µg or 600 IU daily)^(13,31)^. Thirdly, less than a third of this lower recommended intake is met through diet in UK children^(29)^ due to the lack of widespread mandatory food fortification^(28,32)^. Last but not the least, the uptake of vitamin D supplements in UK infants and children is also very low at < 20 %^(33)^ due to poor policy implementation^(34)^. Hence, the UK has a higher prevalence of vitamin D deficiency compared with other countries at comparable latitude with similar proportions of BAME population^(16)^. Robust vitamin D supplementation of all high-risk groups has no notable side effects and is a safe option irrespective of the link. At-risk individuals should be reminded of the need for supplementation at each healthcare contact^(34)^.

Our study was limited by the small sample size given the rarity of the condition. Nonetheless, the data provided are of paramount public health importance. The chronicity of vitamin D deficiency was not known due to the unavailability of parathyroid hormone levels and the concurrent presence of hypophosphatasemia related to the acute insult^(35)^. However, the prevalence of deficiency reported here mirrors data from other studies of predominantly BAME populations^(36,37)^, yet again highlighting the importance of supplementing high-risk individuals and adoption of long-term strategies such as food fortification, as a cost-effective way of optimising population vitamin D status^(38)^.

### Conclusions

PIMS-TS has seen an over-representation of children from BAME background, who are also at greatest risk of vitamin D deficiency. In view of the high prevalence of vitamin D deficiency in our PIMS-TS cohort, we call for mandated, year-round vitamin D supplementation of all high-risk children and adolescents. Food fortification with vitamin D should be strongly considered as a long-term strategy.
